# Risk Perception Among Decision-Makers in the Dominican Republic’s National System for Prevention, Mitigation, and Response to Climate Change-Related Events

**DOI:** 10.3390/ijerph23050565

**Published:** 2026-04-27

**Authors:** Juan Cesario Salas-Rosario, Yanelba Elisa Abreu-Rojas, Antonio Torres-Valle, Ulises Javier Jauregui-Haza

**Affiliations:** 1 Área de Ciencias Básicas y Ambientales, Instituto Tecnológico de Santo Domingo (INTEC), Santo Domingo 10602, Dominican Republic; juan.salas@defensacivil.gob.do (J.C.S.-R.); y.abreu@grupoalfama.com (Y.E.A.-R.); antonio.torres@intec.edu.do (A.T.-V.); 2 Oficina de la Defensa Civil, Santo Domingo 10304, Dominican Republic; 3 Grupo ALFAMA, Construcción y Proyectos Sostenibles, Santo Domingo 10304, Dominican Republic; 4 Instituto Superior de Tecnologías y Ciencias Aplicadas (InSTEC), Universidad de la Habana, La Habana 10400, Cuba; 5 Cátedra UNESCO de Cambio Climático, Resiliencia y Sistemas Complejos, Instituto Tecnológico de Santo Domingo (INTEC), Santo Domingo 10602, Dominican Republic

**Keywords:** adaptation, climate change, decision-makers, environmental sustainability, mitigation, prevention, risk perception

## Abstract

**Highlights:**

**Public health relevance—How does this work relate to a public health issue?**

- The study examines how systematic underestimation of climate-related risks among national decision-makers may weaken preparedness and response in vulnerable Dominican Republic communities.
- By analyzing subjective risk perception, the research reveals gaps that directly affect population safety, disaster risk reduction, and environmental health resilience.

**Public health significance—Why is this work of significance to public health?**

- Findings show critical deficits in understanding climate risks (e.g., low COMP, INVO, INST, CLIM), which are essential for effective governance of climate impacts on health, infrastructure, and livelihoods.
- The work provides a validated diagnostic tool (risk perception profiles using RISKPERCEP) that can help public health institutions identify cognitive and organizational barriers that hinder climate adaptation.

**Public health implications—What are the key implications or messages for practi-tioners, policy makers and/or researchers in public health?**

- Strengthening climate risk literacy and institutional trust among decision-makers can significantly improve anticipatory action, early warning systems, and resource allocation, reducing health and social vulnerabilities.
- Results point to the need for targeted training, updated protocols, and ongoing perception audits to ensure that public health, disaster management, and climate policy operate with accurate and proactive risk understanding.

**Abstract:**

Sustainable development results from the harmonious integration of economic growth, social equity, and environmental sustainability. Building on available risk analysis capacities, this study employs risk perception as a diagnostic tool to evaluate the adequacy of decision-making regarding environmental sustainability in vulnerable human settlements under a changing climate in the Dominican Republic. Using the perceived risk profile approach and a specially designed questionnaire, the research explores issues related to climate change and sustainability, targeting a population composed of decision-makers and professionals engaged in risk assessment. The findings reveal a systematic underestimation of risk across most perception variables, as well as a generally low collective risk perception. The study’s methodological framework enables the identification of proactive measures to strengthen knowledge and performance among decision-makers and stakeholders involved in advancing sustainable development in Dominican human settlements.

## 1. Introduction

Sustainable development has been widely examined in the literature, particularly in relation to global climate change [1,2]. One of the most prominent conceptual frameworks is the “trilemma,” which requires balancing economic growth, social equity, and environmental sustainability [3].

Societies, in their interaction with the environment and governance systems, often face normative contradictions in the design of legislation, manifesting in ongoing conflicts between environmental protection laws and those promoting economic and social development. Reconciling these competing interests remains a major challenge for governments and societies alike [4].

Risk studies have emerged as a valuable approach to assessing sustainability, demonstrating their capacity to encompass both objective and subjective dimensions of analysis [5].

Among the most complex areas of study is risk perception, or subjective risk, which is at a relative disadvantage compared to objective risk [6]. Objective risk can be assessed using event statistics, production records, imaging systems, and other data sources [7,8], often with a reactive focus on impacts. Prospective approaches, such as risk matrices or failure mode and effects analyses, have further advanced objective risk evaluation [9,10].

Risk perception is a fundamental psychological construct that explains how individuals and societies evaluate and respond to threats and hazards. Psychological models addressing risk perception include heuristic and cognitive bias theories, the psychometric paradigm, cultural theory of risk, social amplification of risk, and the concept of risk as feeling. From the perspective of heuristic theories, it is posited that people rely on mental shortcuts, or heuristics, to simplify decision-making under uncertainty [11].

The psychometric paradigm, which is central to this research, is a quantitative approach that seeks to identify the qualitative characteristics of risk that shape people’s perceptions. This paradigm has demonstrated that, beyond the probability of harm, factors such as the dread a risk induces, the degree of perceived control, the unknown nature of the risk, and its catastrophic potential are key determinants [12,13].

Cultural theory of risk argues that risk perceptions are deeply rooted in cultural values and ways of life. The theory highlights how different “ways of life” (hierarchical, individualist, egalitarian, fatalist) tend to emphasize and worry about distinct types of risks [14]. The issue of cultural and religious diversity has a broad impact on risk perception. In this regard, studies such as those conducted by Farrokhi et al. [15] show the relationship of these patterns with health and cognitive dimensions, as well as with lived experiences of extreme climate events. Without delving into the cultural approach to risk perception, and through the psychometric paradigm, the highlighted aspects have been incorporated into our research.

The social amplification of risk explains how risk events and inherent risk characteristics interact with psychological, social, institutional, and cultural processes, amplifying or attenuating individual and societal perceptions of risk. This amplification occurs in two main stages: in the transfer of information about the risk and in the mechanisms of societal response [16].

The concept of “risk as feeling” emphasizes the crucial role of emotions and affect in decision-making and risk perception. This concept suggests that individuals perceive and act upon risk in two fundamental ways: “risk as analysis,” which involves logic and reason, and “risk as feeling,” which refers to instinctive, intuitive, and automatic reactions to danger [17].

Risk perception—understood as a specific dimension of risk—is intrinsically linked to all aspects of sustainability, since governance structures and communities, through their decisions and participation, act simultaneously as promoters and recipients of risks arising from the economy–environment–society nexus.

The persistent gap between technical assessments (objective risk) and public perceptions underscores the need to move beyond a “knowledge deficit” model, which assumes that the public lacks information [13]. A more effective approach recognizes the legitimacy of public concerns, which often incorporate ethical, moral, and emotional dimensions absent from technical analyses [16].

Risk perception, in contrast with the objective risk, measures how individuals or social groups think about or are aware of specific hazards that accompany society in its daily functioning. These perceptions shape behaviors toward risks. Consequently, research has increasingly advanced toward perceptual and behavioral measurement, forming the basis for subjective risk assessments [18,19,20,21,22].

One of the most successful methodologies in recent years has been the use of perceived risk profiles [5,18,19,20]. While profiles can be constructed directly from survey results [21,23], the use of variable-based profiles has gained traction due to their integrative capacity. The article presents the use of the psychometric paradigm (based on variables and surveys), computerized through the RISKPERCEP software (version 2025) [24,25,26]. Each variable, encompassing the results of multiple questions, provides a clearer view of group risk perception, serving as a tool to aggregate and average opinions [26]. When the subjects of study are decision-makers in activities associated with risk, it is assumed that they are trained and specialized in relevant topics. This implies that their risk perception should be shaped by their professional knowledge [27].

As an example, the variables employed to measure risk perception include individual characteristics (personal involvement, knowledge about the risk, uncertainty), those related to physical risk (catastrophism, past history of events), and those associated with risk management (risk–benefit balance, trust in institutions). After consulting specialized references on the role of risk managers, knowledge gaps have been identified, particularly contradictions between the perceptions of decision-makers and those of the public receiving risk management. A summary of the aspects that generate conflicts in decision-making, organized according to the consulted references, is presented below:

- Decision-makers or managers should be characterized by their personal involvement in the situation and by their experience with the risk under management [28].
- Managers should converge with the physical space and temporal dimension of risk recipients and adopt pro-environmental positions [29].
- Managers must address the limits of societal adaptation to losses and damages [2].
- Managers should be aware of the possible existence of multi-hazard catastrophism and maintain proximity to the problem to be solved [30].
- Decision-makers are responsible for resolving the conflict between uncertainty and risk–benefit (risk aversion) and the knowledge of past disaster history combined with catastrophism (loss aversion). This is supported by Kahneman and Tversky’s prospect theory [31]. In the same vein, other authors confirm the existence of contradictions between losses and pure experiences, which may condition managerial decisions [32].
- Additional issues coinciding with these conflicts include the importance of government proximity to disasters and their management, the incorporation of disasters into societal education, coordinated work between local governments and communities, and the unevenness of public risk perception [33].
- Decision-makers must also consider the potential low trust in institutions, as well as differences in perception by place of residence, gender, educational level, and income [34,35].
- Findings from some studies show the relationship between respondents’ socioeconomic characteristics and their political orientation. For example, Paniello-Castillo et al. [36] report that women, low-income individuals, and those with left-leaning political tendencies perceive higher risks across all hazards compared to men, higher-income individuals, and those with right-leaning tendencies [37]. Younger individuals perceive higher risks for climate change and the economic crisis but lower risks for epidemics compared to older individuals. These findings highlight the importance of tailored communication strategies to address diverse perceptions and enhance public support for climate policies.

Finally, it is concluded that risk management for decision-making must be multifactorial, and the most effective roles are achieved when the management team has first-hand knowledge of the risks perceived by recipients, their associated catastrophism, uncertainty, risk–benefit considerations, past disaster history, and the unevenness of perception linked to residence, gender, educational level, and income. Equally important are the adoption of pro-environmental positions, the valuation of societal education on disasters, and coordination between local governments and communities. In this way, the research focuses on exploring the gap that arises from decision-makers’ lack of knowledge regarding the enumerated issues, whether from the perspective of their own decisions and/or the implementation of associated management.

The objective of this research is to employ risk perception as a diagnostic tool for evaluating the adequacy of decision-making among actors within the Dominican Republic’s national system for prevention, mitigation, and response to climate change-related events.

## 2. Materials and Methods

The methodology selected for this study was the perceived risk profile approach, which relies on the use of variables and structured surveys. A key element in applying this methodology was the availability of specialized software designed for risk perception studies. The RISKPERCEP code, developed by an interdisciplinary team at the University of Havana, Cuba, has been successfully applied in multiple investigations on occupational risk perception and public risk perception [25,38,39].

Within the field of occupational risks, perception studies have encompassed diverse groups, including personnel from clinical laboratories, veterinary services, vaccine production facilities, dental clinics, and telecommunications linemen, among others [24,38].

**Figure 1:**
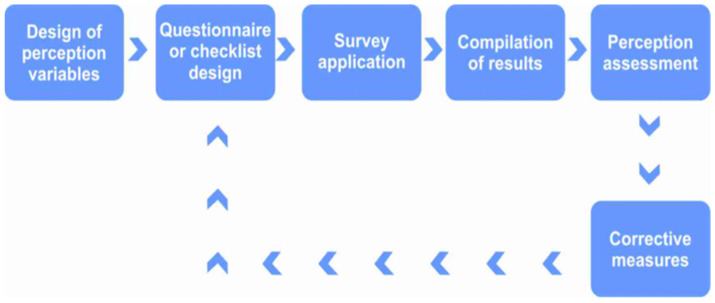
Algorithm for Risk Perception Study (modified from [25]).

In the public sector, perception studies on climate change have proven particularly relevant. Notable examples include research conducted among teachers in UNESCO-associated schools in Cuba and among university professors in Honduras [24,39]. Additional studies have examined risk perception related to the labor impacts of the COVID-19 pandemic, with international scope [25]. The algorithm describing the execution of this study is presented in Figure 1.

The design of risk perception variables (Table 1) depends on the objectives of the study [18,19,20]. For instance, in the analysis of psychosocial risks, variables are typically grouped into three categories: individual-related variables, variables associated with the physical nature of the risk, and variables linked to risk management [5,20].

The determination of the variables considered in this research was complemented by an in-depth study of issues related to climate change and sustainability, enabling a multidisciplinary analytical approach. Within this framework, all survey questions can be regarded as directly or indirectly linked to climate change, while approximately 50% specifically address environmental sustainability.

In this regard, several authors have emphasized the importance of decision-makers and managers being personally involved (personal involvement) and possessing experience (familiarity, past event histories) and knowledge (risk comprehension) regarding the situation under their management [28,31]. Equally relevant are the levels of catastrophism (losses, catastrophism, immediacy of consequences, identification with victims) associated with risks [2,30,32], as well as demographic variables of perception such as gender, age, educational level, and income [33,34,35,36]. These demographic variables are not explicitly listed in the framework presented but are the subject of further study in this research. Additionally, some management-related variables identified by other authors [1,25,39] have been incorporated into the study—such as benefits, risk–benefit inequity, organizational climate, trust in institutions, and the role of the press—given their importance from the perspective of risk management and communication.

An important aspect of variable selection was the analysis of their relationship with associated risk perception. Some variables exhibit direct proportional behaviors—such as catastrophic potential, panic generated, and immediacy of consequences—while others behave inversely, including familiarity with risk, perceived ability to control it, and reversibility of consequences. The variable understanding of risk (COMP) presents a particular case of extreme behavior, as both experts and non-specialists tend to underestimate it equally. To avoid introducing subjectivity, all variables were treated as independent and assumed to contribute equally to quantification.

The survey design followed expert guidelines. Questionnaires were tailored to the types of hazards and study groups, structured to foster empathy, and organized to progress from the familiar to the uncertain, from general to specific, and from institutional to individual perspectives [40].

For evaluation purposes, most questions were closed-ended, with responses ordered on a unidirectional scale of three levels: 1 = risk underestimation, 2 = adequate estimation, 3 = risk overestimation. Additional question formats, such as ranking and multiple-choice, enriched the survey. These required a specific evaluation key to convert responses into the original three-level scale.

It is important to note that the approach based on responses classified into three levels may appear simple, yet it is sufficient to study directional bias toward underestimation or overestimation of risk in relation to an external reference, rather than through continuous and detailed deviations. Both classical and contemporary studies on unrealistic optimism and comparative risk perception adopt similar categorical distinctions to capture systematic cognitive biases (e.g., [41,42,43]).

The three-level scale applies to variables with direct evolution relative to risk perception. For variables with inverse or extreme behavior, the software tool automatically adjusted values during evaluation. The survey applied in this study is presented in File S1 (Supporting Information), showing the relationship between each question and its corresponding variable, as well as the evaluation keys used. Surveys were conducted individually using prepared templates.

The surveys were conducted during consultative meetings with decision-making personnel from different hierarchical levels of Civil Defense, members of the National System of Prevention Mitigation Response and their collaborators. Participation in the survey was voluntary, and the selection of respondents depended on their active involvement in the disaster management process, which constitutes part of their institutional duties. Data collection was conducted through KoboToolbox applications that compiled and organized the information gathered during the meetings, producing input files for the RISKPERCEP software (version 2025), which was subsequently used to carry out the risk perception assessment.

### 2.1. Sample Size Determination

Sample size was calculated using the following expression, embedded in the RISKPERCEP system [44]:

$$n=\frac{NZ^{2}pq}{e^{2}\left(N=1\right)+Z^{2}pq}$$

where

***n*** = sample size

***p*** = probability of occurrence or expected proportion

***q*** = probability of non-occurrence

***e*** = precision (maximum admissible error in terms of proportion)

***Z*** = probabilistic factor as a function of confidence level

***N*** = population size

Based on response probabilities of 0.3 (success) and 0.7 (failure), a confidence level of 95%, and a precision of 6%, the required sample size was calculated as 58 respondents, representing a population of 5000. In practice, 60 individuals were surveyed, satisfying the requirement.

The sample comprised 44 men and 16 women, distributed across age ranges as follows: 4 between 18 and 30 years, 24 between 31 and 45 years, 20 between 46 and 60 years, and 12 over 60 years. Of these, 35 were decision-makers and 25 were risk management technicians.

### 2.2. Data Compilation and Processing

The survey consisted of 27 questions (Supporting Information). Results were compiled into a matrix of 27 columns × 60 rows: the first column identified respondents, while the remaining 27 stored their responses (scored 1–3, with 0 for non-response). Each row represented one respondent’s dataset.

Data were initially processed in Excel tables, which facilitated automated input into the RISKPERCEP code. Excel also served as an intermediate step for applying the evaluation keys described above.

Risk perception evaluation was based on quantification indicators expressed through simple schemes, enabling averaged assessments at the variable, respondent, and group levels [25]. Results were presented both analytically (tables) and graphically (histograms and line plots).

Since the system employs ordinal qualitative values converted into numerical scores, subsequent averaging is statistically non-standard. To address this, an instrumental license was adopted, defining a new non-statistical estimator termed the Weighted Risk Perception Score (hereafter, Score). Dispersion values were also calculated for each variable relative to its score, illustrating group consensus and collective tendencies.

Ultimately, during the survey completion process, each individual indicated their response options, which, during data collection, were assigned values (0 if unanswered, or between 1 and 3 according to their choice). These values were compiled into the database managed by KoboToolbox. It is important to note that average values are hereafter referred to as Score. The evaluation of each perception variable at the individual level results from the Score of the questions contributing to it, while the assessment of each individual’s overall perception corresponds to the Score of their respective individual variables. Once all surveys were completed, the group-level process included the Score of the evaluations of the individual variables of all respondents, thereby providing assessments of variables at the group level, while group perception was evaluated through the Score of variables calculated at that level. In calculating group-level variables, a statistical study of dispersion relative to the Score of each variable was also incorporated. Corrective measures were derived from the interpretation of results, guided by the Pareto principle to identify the most influential contributors (variables, respondents, and groups) and prioritize tasks with the greatest impact. Risk perception should be reassessed after implementing corrective measures to verify their effectiveness.

## 3. Results

The evaluation of risk perception among the surveyed group is presented in Table 2.

**Figure 2:**
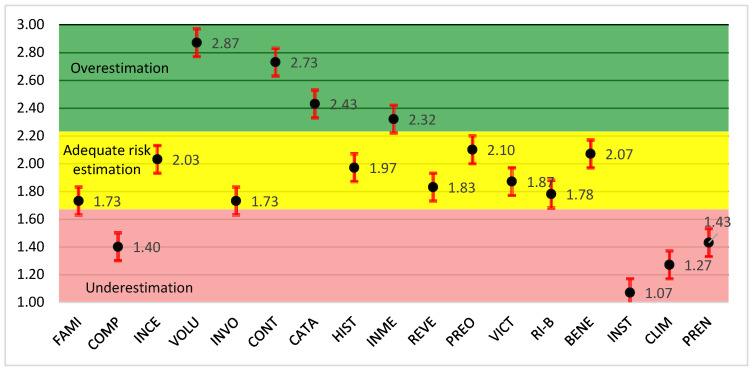
Perceived risk profile for the study on environmental sustainability in human settlements of the Dominican Republic (FAMI—familiarity with risk, COMP—understanding of risk, INCE—uncertainty, VOLU—voluntariness, INVO—personal involvement, CONT—ability to control, CATA—catastrophic potential, HIST—past history of disasters or hazards, INME—immediacy of disasters/consequences, REVE—reversibility of consequences, PREO—concern, VICT—identity of victims, RI-B—risk–benefit inequality, BENE—benefits of exposure, INST—trust in institutions, CLIM—organizational climate, PREN—role of the press or broadcast media). The dimensionless risk perception scale (Y-axis) indicates the following: (1–1.67)—underestimation of risk; (1.68–2.23)—adequate risk estimation; and (2.24–3)—overestimation of risk.

The overall average risk perception for the group was 1.95, which is close to adequate estimation (with 2 being the benchmark for proper risk assessment). However, significant deficiencies were observed in several variables, particularly HIST (past history of disasters), INME (immediacy of consequences), RI-B (risk–benefit inequity), and BENE (benefits). In addition, the variable PREN (role of the press) exhibited high dispersion, indicating a lack of consensus among respondents regarding the media’s role in communicating climate-related risks. The graphical representation of the perceived risk profile is shown in Figure 2.

In general, the variables most responsible for the underestimation of global risk in both evaluation approaches were a low understanding of risk (COMP), low perceived personal involvement (INVO), limited trust in institutions (INST), and weak organizational climate (CLIM).

### Demographic Analysis

**Figure 3:**
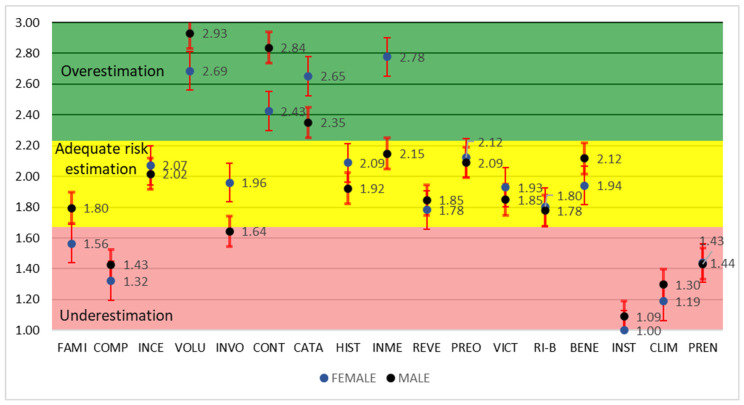
Comparative risk profile by gender for the study on environmental sustainability in human settlements of the Dominican Republic (FAMI—familiarity with risk, COMP—understanding of risk, INCE—uncertainty, VOLU—voluntariness, INVO—personal involvement, CONT—ability to control, CATA—catastrophic potential, HIST—past history of disasters or hazards, INME—immediacy of disasters/consequences, REVE—reversibility of consequences, PREO—concern, VICT—identity of victims, RI-B—risk–benefit inequality, BENE—benefits of exposure, INST—trust in institutions, CLIM—organizational climate, PREN—role of the press or broadcast media). The dimensionless risk perception scale (*y*-axis) indicates the following: (1–1.67)—underestimation of risk; (1.68–2.23)—adequate risk estimation; and (2.24–3)—overestimation of risk.

Among the possible demographic analyses, only gender was retained (Figure 3), since no significant differences were observed in terms of educational level or age within the surveyed population.

The comparison of risk perception with respect to the variable of immediacy of consequences revealed an expected pattern of risk overestimation among women, with similar tendencies—though less pronounced—observed in the variables of personal involvement, catastrophism, and victim identification. These aspects have likewise been reported by other authors [36,45,46].

Differences in average levels of risk perception by gender were not statistically significant. However, a slight distortion was observed in the comparison of mean risk perception between genders, with male respondents showing marginally higher values. This outcome may be attributed to the predominance of men in the sample compared to the relatively small female population. The issue of sample size imbalance could not be resolved within the scope of this study.

## 4. Discussion

It is acknowledged that classifying survey responses into three levels constitutes a simplification and may sacrifice certain information compared to continuous measures. Nevertheless, given the objectives of the present study—namely, to identify systematic patterns of misperception and their implications for public health—it is considered that the benefits of conceptual clarity, comparability with previous research, and ease of interpretation outweigh the limitations [47,48,49,50].

The reasons underlying the underestimation of risk are reflected in the generally incorrect responses to questions associated with the key variables driving this outcome.

With respect to understanding of risk (COMP), respondents demonstrated misinterpretation of the relationship between climate change and its anthropogenic causes; erroneous assumptions regarding the link between atmospheric chemical composition and industrial development; lack of knowledge about greenhouse gases (GHGs) and their sources; inadequate identification of the components of energy sustainability; incorrect comprehension of global hazards associated with rising average atmospheric temperatures; and limited awareness of the potential role of technologies in reducing GHG emissions.

Regarding personal involvement (INVO), respondents failed to recognize the role of families in climate change, as well as the indirect impacts of individual and household practices that exacerbate extreme events. Institutions were also perceived as failing to adequately fulfill their role in controlling the impacts of climate change. Moreover, they were not seen as fostering an appropriate organizational climate (CLIM) in relation to activities linked to climate risk management, including energy, economy, transportation, food security, and the protection of people and nature.

Risk perception has been a recurring theme in IPCC reports [1,2,51], as the cognitive frameworks of communities and decision-makers are critical factors influencing the acceptance or rejection of governance policies—particularly adaptation and mitigation measures aimed at addressing this global phenomenon. Raising awareness of these issues, together with the timely adoption of measures, can serve as a counterbalance to the negative contributions of human activity to the survival prospects of future generations.

Two previous studies on climate change risk perception were used to support this application to energy sustainability, the UNESCO-sponsored study on climate change risk perception in the Cuban education sector [39] and the study promoted by Zamorano University among Honduran universities [24]. In the first case, the methodology was applied to a population shaping Cuban society, with the aim of adapting educational plans to the diagnostic outcomes of the analysis. The study encompassed 47 Cuban schools associated with UNESCO, where a specialized survey was administered to 75 administrators and 1061 teachers [5,39]. In the second case, with similar objectives, a specialized questionnaire was applied to 457 professors from Honduran universities [24]. For the present application, only the questions and variables relevant to energy sustainability were extracted from these prior studies [24,39]. The variables considered included ability to control risk (CONT), through survey items on state positions regarding climate change; understanding of risk (COMP), considering concepts of sustainable development and theoretical/methodological preparedness on climate change; catastrophic potential (CATA), exploring types and levels of climate change impacts; reversibility of consequences (REVE), addressing the role of states in policy design; benefits (BENE) and risk–benefit inequity (RI-B), examining the trade-offs of technological development against environmental damage; and organizational climate (CLIM), assessed through questions on curricular content, databases, and materials available on climate change, adaptation, mitigation, and sustainable development.

There is a clear convergence between the findings of this study and those reported by [24], particularly for the variables COMP (understanding of risk), RI-B (risk–benefit inequity), CLIM (organizational climate), and BENE (benefits). In all these cases, the variables consistently underpin the underestimation of risk.

Other reference studies were also consulted [5], which incorporated the opinions of groups not specialized in energy sustainability. These studies addressed aspects that, directly or indirectly, encompassed environmental sustainability, social equity, and energy security, thereby supporting the criteria advanced by Samanes [52]. Comparison with these cases revealed variables similar to those identified as responsible for risk underestimation in the present research.

Reports from the Intergovernmental Panel on Climate Change [1] highlight several relevant statements on risk perception in the context of climate change, underscoring the utility of the approach proposed in this study. Examples include the following:

- “Climate change involves complex interactions and behavioral changes across diverse impacts. The focus on risk, which is new in this report, supports decision-making in the context of climate change and complements other elements of the assessment. People and societies may perceive or prioritize risks and their potential benefits differently, depending on specific values and goals.”
- “Adaptation planning and implementation at all levels of government must be consistent with social values, objectives, and perceptions of risk (high confidence). Recognition of diverse interests, circumstances, sociocultural contexts, and expectations can enhance decision-making processes.”
- “The design of climate policy is influenced by how individuals and organizations perceive risks and uncertainties, and how they account for them. People often rely on simple decision rules, such as preference for the status quo. Individuals and organizations differ in their degree of risk aversion and in the relative importance they assign to immediate versus delayed issues. As a complement to formal methods, policy design can be improved by considering risks and uncertainties in natural, socioeconomic, and technological systems, as well as decision processes, perceptions, values, and resources.”
- “Options and outcomes of adaptation measures must reflect divergent resources and capacities, as well as multiple interaction processes. Measures are framed as trade-offs among prioritized values, competing objectives, and different visions of development that may evolve over time. Iterative approaches allow development pathways to integrate risk management, enabling consideration of diverse policy solutions, since risk and its measurement, perception, and understanding evolve over time.”

These elements demonstrate the strong relationship between risk perception and the broader challenges of climate change.

In 2022, the Dominican Republic presented its study on knowledge and perceptions of climate change, conducted through a specialized survey designed by the United Nations. Overall, the results revealed a relatively high level of understanding of climate change compared to other countries, particularly among individuals with postsecondary education. This study was extended to 115 countries worldwide.

Although these are not specialized references on risk perception among decision-makers or managers of climate risk, this study reflects a cultural and knowledge pattern worth highlighting [21,23]. With regard to the Dominican public, it was found that

- A total of 92% surveyed believe that climate change affects their daily lives.
- A total of 93% support stricter government measures requiring behavioral changes to address climate change.
- A total of 85% believe that government priorities should focus on the environment and sustainable growth rather than economic growth at any cost.
- A total of 82% perceive climate and environmental policies as improving daily comfort (e.g., food, health).
- A total of 78% believe such policies will generate economic growth and wealth, while 70% expect them to create more new jobs than those lost.
- A total of 67% report that climate change affects their income or livelihood
- A total of 60% have already experienced water scarcity (e.g., shortages or conflicts over water resources).
- A total of 51% believe they may need to relocate to another region or country due to climate change.

Regarding energy options, the study highlighted the following:

- A total of 82% advocate for investment in renewable energy sources.
- A total of 52% prefer large-scale renewable projects (hydroelectric, wind, solar, geothermal), while 30% favor small-scale solutions (rooftop solar panels, small hydro plants). This overwhelming preference reflects growing awareness of sustainability and the role of clean energy in combating climate change.
- A total of 70% recognize human activities, such as fossil fuel use, as the primary causes of climate change—an essential factor for public support of emission reduction policies.
- In terms of individual energy savings, economic reasons were the main driver rather than environmental awareness: 85% reported adopting energy-saving measures due to the current economic crisis and household expenses.
- A total of 27% believe climate change is a natural process caused by phenomena such as solar variability or volcanic activity, compared to a global average of 19%.

The central contribution of our study does not lie in its geographic specificity but rather in its analytical framework, target population, and diagnostic use of risk perception, all of which are broadly applicable in comparable institutional and socio-ecological contexts. First, the population under study—decision-makers and technical personnel involved in national disaster risk management systems and climate governance—is structurally comparable across countries, particularly in Small Island Developing States (SIDS), Latin America and the Caribbean, and other climate-vulnerable regions. The international literature consistently shows that the systematic underestimation of climate-related risks by institutional actors is not country-specific but a recurrent pattern linked to cognitive biases, institutional cultures, and organizational constraints [13,30].

The variables identified as key factors in risk underestimation in our study—limited risk comprehension (COMP), low perception of personal involvement (INVO), weak trust in institutions (INST), and deficient organizational climate (CLIM)—closely reflect findings from studies conducted in other national contexts. Similar perception deficits have been documented among decision-makers or key actors in Greece [22], Turkey [34], Honduras [24], China [6], and deltaic environments in Europe [30]. This convergence suggests that the observed patterns are structurally and psychologically grounded rather than context-dependent anomalies.

The methodological approach based on the psychometric paradigm and risk perception profiles derived from variables, implemented through the RISKPERCEP system, has already been successfully applied in multiple countries and sectors, including education, occupational health, public risk communication, and climate change adaptation [18,25,39]. This demonstrates that the analytical structure and scoring logic are transferable, enabling replication and comparative analysis in other national or institutional contexts. Beyond its generalizability, this study contributes to the literature in several substantive ways:

- Focusing on decision-makers rather than the general public: Few studies systematically assess risk perception among institutional decision-makers, despite their crucial role in activating early warning systems, allocating resources, and implementing adaptation and mitigation policies. Our work directly addresses this gap, aligning with recent calls in the IPCC AR6 and disaster risk governance research to better understand institutional cognition and behavior.
- Integration of risk perception with sustainability and governance: The study advances existing research by explicitly linking risk perception variables with environmental sustainability, organizational climate, and governance performance, moving beyond isolated attitudes toward a system-oriented diagnostic perspective. This integrative approach complements recent work published in the *International Journal of Disaster Risk Reduction*, *Risk Analysis*, and *Sustainability*.
- Operational diagnostic tool relevant for policy: Rather than treating risk perception as a descriptive outcome, the study demonstrates its use as a practical diagnostic instrument capable of identifying cognitive and organizational bottlenecks that weaken preparedness and anticipatory action. This operational orientation enhances the study’s relevance for policymakers and practitioners internationally, particularly in countries implementing the Sendai Framework, National Adaptation Plans (NAPs), and Nationally Determined Contributions (NDCs).

In summary, although the empirical setting is national, the identified patterns, theoretical foundation, and methodological approach support a cautious yet meaningful generalization, especially to other climate-vulnerable countries and institutional risk governance systems. We believe this strengthens the manuscript’s relevance and its contribution to the international literature on climate risk perception and public health resilience.

### 4.1. Methodological Considerations Regarding Survey Processing and Gender Differences

A limitation of these studies [21,23] is their reliance on independent questions rather than a structured set of perception variables, which disperses conclusions and complicates the formulation of targeted measures. Nevertheless, there are evident similarities between the topics addressed in these surveys and those explored in the present study. Although efforts were made to ensure demographic representativeness (age, specialization), material and time constraints may have introduced bias that was not exhaustively analyzed. In terms of gender, the variables INVO (personal involvement), CATA (catastrophic potential), HIST (past history of disasters), and INME (immediacy of consequences) showed slight overestimation among female respondents. This tendency is attributed to women’s traditionally protective role within households and broader gender norms in society [24,45]. Traits such as prudence and vulnerability, coupled with the pursuit of safe environments, have historically been associated with women [24,46]. These factors may lead to safer, less risk-prone behaviors. Additionally, gender-based violence places women in situations of uncertainty and vulnerability, fostering more cautious and critical attitudes toward their environment and, consequently, higher risk perception.

Future research could be strengthened by incorporating personality assessments of respondents [25,53], which were not included in this study to simplify data collection.

### 4.2. Implications

It is evident that greater emphasis must be placed on preparing respondents on topics related to climate change and associated risks. Such preparation is essential to ensure their effective role in decision-making processes regarding sustainability in human settlements in the Dominican Republic under changing climate conditions.

### 4.3. Actions to Address Risk Perception Among Decision-Makers in the Dominican Republic’s National System for Prevention, Mitigation, and Response to Climate Change-Related Events

At the institutional level, the Dominican Republic has a robust legal and regulatory framework (Law 147-02; a National Emergency Commission as the coordinating body; the National Comprehensive Risk Management Plan approved by Decree 275-13; hydrometeorological plans/protocols; and an interinstitutional Early Warning System, SAT). However, it requires strengthened capacities, improved data governance, and enhanced SAT operations to enable more timely, evidence-based decisions.

In terms of climate policy, the National Adaptation Plan for Climate Change 2015–2030 and the ongoing National Adaptation Plan (NAP) process provide a roadmap for adaptation. Meanwhile, the Nationally Determined Contribution (NDC) 2020 and its Action Plan 2022–2025 set targets and axes (e.g., water security, resilient cities, health, coasts/tourism) that demand adequate risk perception among decision-makers for effective implementation. The international literature supports the notion that risk perception conditions policy acceptance and the speed of action; both the IPCC AR6 and the Sendai Framework emphasize risk governance, understanding, and preparedness (priorities 1–4). Global surveys also show strong public support for climate measures in the Dominican Republic, representing an opportunity to align public and technical decision-making.

To elevate risk perception among decision-makers within the National System for Prevention, Mitigation, and Response (SN-PMR), correcting cognitive and organizational biases and aligning decisions with SAT and scientific evidence—thus reducing reaction time and prioritizing adaptation, preparedness, and communication measures consistent with PNGR, PNACC/NAP, and NDC—the following actions are proposed:

1. Institutionalize a risk-informed decision principle (hazard–exposure–vulnerability), prioritizing anticipatory action when SAT projects significant impacts, with technical justification documented for each activation.
2. Train decision-makers in climate science (IPCC AR6), local scenarios and impacts, iterative risk management, SAT action thresholds, risk ethics, cognitive biases (normalcy, status quo, discounting the future, illusion of control), and multi-hazard decision simulations.
3. Develop an interinstitutional protocol with checklists by alert level (green/yellow/red), responsibilities, maximum response times, and anticipatory “packages” (pre-positioning, drainage clearance, segmented communication), particularly for local-level decision-makers.
4. Define minimum indicators: percentage of decisions activated by SAT thresholds; activation time relative to norms; percentage of drills executed; checklist compliance; improvement of COMP/INVO by institution; interinstitutional satisfaction. Link KPIs to performance evaluations and non-salary incentives.
5. Establish decision traceability systems (hazard–exposure–vulnerability matrix) aligned with IPCC AR6 and Sendai, to strengthen transparency and organizational learning.
6. Activate the national prevention, mitigation, and response fund under Law 147-02 to finance disaster risk management measures, both anticipatory and responsive.
7. Conduct periodic risk perception audits to measure gaps between objective risk and prior perception, track profile evolution, and set improvement targets.
8. Include post-disaster evaluations of anticipation errors and detected biases, feeding back into SAT protocols, KPIs, and training content.
9. Parameterize each SN-PMR activation/decision with prioritized adaptation measures (water, cities, health, coasts, ecosystems), reporting co-benefits and advancing adaptation monitoring through the SN-PMR and PNACC/NAP/NDC traceability matrix.

### 4.4. Study Limitations

This study is not exempt from limitations commonly associated with risk perception research. Methodologically, the assumption of independence among variables within the perceived risk algorithm limits exploration of inter-variable relationships. Fragmenting perception into individual variables increases subjectivity in interpretation. While variables could be further subdivided or expanded, this would undermine survey pragmatism and introduce additional complexity.

Another limitation concerns the number and type of questions used to investigate each variable. For practical reasons, and considering the target population, the number of questions was limited. Fewer questions per variable inevitably reduce the depth of analysis, contributing to subjectivity in results.

The closed-question format facilitated mathematical processing but restricted the incorporation of new insights derived from respondents’ creativity and knowledge. Processing open-ended responses remains challenging, though artificial intelligence algorithms may offer innovative approaches not included in this study. Finally, extrapolating results to practical applications in risk management and communication policies is a critical aspect that must be acknowledged.

## 5. Conclusions

This study demonstrates the low risk perception among personnel involved in decision-making regarding the environmental sustainability of human settlements in the Dominican Republic under changing climate conditions. It is therefore essential to implement a knowledge update cycle to ensure their effective role as decision-makers in this domain. The main variables responsible for underestimating sustainability-related risk are as follows:

- Low understanding of risk (COMP) regarding the relationship between energy and climate change;
- Low perceived personal involvement (INVO) concerning the individual and family role in energy sustainability and the indirect impacts of energy use on society;
- Low trust in institutions (INST) regarding their role in environmental management;
- Weak organizational climate (CLIM) in institutions and enterprises, limiting the adoption of low-impact environmental measures.

This research proposes a tool informed by prior studies on climate change risk perception but specialized in exploring issues of environmental sustainability in human settlements. Although direct comparison with similar studies focused on human settlements is not possible, the findings demonstrate clear convergences with other investigations addressing climate change, whether through variable-based approaches or isolated survey questions.

## Figures and Tables

**Table 1:** Variables employed in the study of risk perception among decision-makers.

No.	Code	Description	Topics Covered in the Survey (Number of Questions)
Individual-Related Variables			
1	FAMI	Familiarity with risk—Degree of experience of the subject with the situation	Frequency of exposure to extreme events (1)
2	COMP	Understanding of risk—Level of knowledge of the individual regarding the risk	Origin of climate change, atmospheric composition, greenhouse gases (GHG), sources of GHG, concept of energy sustainability, threats associated with global warming, technologies for reducing GHG emissions (6)
3	INCE	Uncertainty—Perception of the extent of scientific knowledge on the subject	Relationship between climate change, human activity, and/or natural variability (1)
4	VOLU	Voluntariness—Degree to which the subject decides whether or not to be exposed to risk	Priority of measures imposed on society to reduce climate change effects, ordered by voluntariness of acceptance (1)
5	INVO	Personal involvement—Extent to which the activity directly affects the individual or their family	Family practices for sustainability, knowledge of climate change impacts on the environment (2)
6	CONT	Ability to control—Extent to which the subject can effectively act to modify the risk situation	Measures to control the effects of extreme events, individual or family actions to reduce environmental impact (2)
**Physical risk variables**			
7	CATA	Catastrophic potential—Severity and concurrence of consequences in space and time	Selection of catastrophic potential for floods, cyclones, landslides, and heavy rainfall (4)
8	HIST	Past history of disasters—Extent to which the activity has a record of previous catastrophes or hazards	Recollection of past climatic events due to their destructive impact on the country (1)
9	INME	Immediacy of consequences—Degree to which consequences occur immediately	Selection of immediacy of consequences for floods, cyclones, landslides, and heavy rainfall (4)
10	REVE	Reversibility of consequences—Extent to which consequences are reversible	Recollection of close victims of extreme events (1)
11	PREO	Concern—Extent to which the event generates emotions such as fear, anxiety, or distress	Psychological impact and preventive measures reflecting concern about extreme climatic events (2)
12	VICT	Identity of victims—Degree of knowledge or family closeness to victims	Recollection of close victims of extreme events (1)
**Risk management-related variables**			
13	RI-B	Risk–benefit inequality—Imbalance between the benefits derived from the risk situation and the costs it generates	Trade-offs between individual comfort and commitments to limiting environmental impact (2)
14	BENE	Benefits of exposure—Inadequate estimation or understanding of the benefits	State support for disaster victims and resource allocation (1)
15	INST	Trust in institutions—Degree to which the individual trusts or gives credibility to the institutions responsible for safety	Role of institutions in reducing environmental impacts of energy use (1)
16	CLIM	Organizational climate—Degree of organization observed in the management aimed at adapting to and mitigating climate change (CC)	Role of the state in addressing the climate crisis in energy, economy, transport, food security, protection of people, and nature (6)
17	PREN	Role of the press or broadcast media—Degree to which the individual trusts the role of the media in informing about the risk in question	Preferred sources of information on climate change initiatives (1)

**Table 2:** Risk perception results of decision-makers. (FAMI—familiarity with risk, COMP—understanding of risk, INCE—uncertainty, VOLU—voluntariness, INVO—personal involvement, CONT—ability to control, CATA—catastrophic potential, HIST—past history of disasters or hazards, IMME—immediacy of disasters/consequences, REVE—reversibility of consequences, PREO—concern, VICT—identity of victims, RI-B—risk–benefit inequality, BENE—benefits of exposure, INST—trust in institutions, CLIM—organizational climate, PREN—role of the press or broadcast media).

Variable	Mean Value (Dispersion)	Variable	Mean Value (Dispersion)
FAMI	1.73 (20, 33, 7)	REVE	1.83 (13, 41, 6)
COMP	1.40 (11, 41, 8)	PREO	2.10 (7, 25, 28)
INCE	2.03 (3, 49, 8)	VICT	1.87 (18, 35, 7)
VOLU	2.87 (8, 52, 0)	RI-B	1.78 (17, 39, 3)
INVO	1.73 (12, 11, 37)	BENE	2.07 (9, 43, 7)
CONT	2.73 (9, 14, 37)	INST	1.07 (0, 56, 4)
CATA	2.43 (11, 28, 21)	CLIM	1.27 (0, 52, 8)
HIST	1.97 (3, 55, 0)	PREN	1.43 (34, 0, 26)
INME	2.32 (16, 23, 20)	Average	1.95 (3, 55, 2)

## Data Availability

The data presented in this study are available on request from the corresponding author.

## Supplementary Materials

The following supporting information can be downloaded at: https://www.mdpi.com/article/10.3390/ijerph23050565/s1. File S1. Questionnaire.

## Abbreviations

The following abbreviations are used in this manuscript:

BENE	Benefits of exposure
CATA	Catastrophic potential
CLIM	organizational climate
COMP	Understanding of risk
CONT	Ability to control
FAMI	Familiarity with risk
HIST	Past history of disasters or hazards
INCE	Uncertainty
INME	Immediacy of disasters/consequences
INST	Trust in institutions
INVO	Personal involvement
PREN	Role of the press or broadcast media
PREO	Concern
REVE	Reversibility of consequences
RI-B	Risk–benefit inequality
VICT	Identity of victims
VOLU	Voluntariness
