# Supplementary material for: Risk Perception Among Decision-Makers in the Dominican Republic’s National System for Prevention, Mitigation, and Response to Climate Change-Related Events
Source: Int J Environ Res Public Health. 2026 Apr 27;23(5):565. doi: 10.3390/ijerph23050565 (PMC13205940; doi:10.3390/ijerph23050565)
Supplement: Supplementary file 1 [file ijerph-23-00565-s001.zip › ijerph-4238003-supplementary.pdf]

Survey on Risk Perception in Human Settlements Vulnerable to Disasters in Greater Santo Domingo

Introduction and Informed Consent

This survey is part of a doctoral research project, which aims to investigate the perception of disaster risk levels in the national territory and establish their relationship with disaster risk management. Completing the survey will take approximately 15 minutes. Participation is voluntary, and you may withdraw at any time.

By participating, you confirm that:

- Your responses will be used for research purposes.
- Your opinions will be treated confidentially.
- You are free to withdraw at any time.
- By answering the questions, you provide consent to participate.

Do you agree to participate in this survey?

- Yes (access to the survey)
- No (Exit questionnaire)

Section I. Participant Profile

- **Sex:** Male / Female
- **Age Range:** Under 18 / 18–30 / 31–45 / 46–60 / Over 60
- **Group Identification:** Citizen / Decision-maker / Disaster risk management technician
- **Highest Academic Level:** Primary / Secondary / Technical or incomplete university / University degree / Postgraduate

Section II. Survey Questions

1. Frequency with which your community is affected by extreme climatic events (per year): Low / Medium / High

2. Causes of climate change (according to your knowledge): Natural causes / Human activities / Both

3. Relationship between atmospheric composition changes and Earth's average surface temperature (Tm): Indirect / Direct / No relationship

4. Greenhouse Gases (GHG): Mark gases you consider GHG; mark with two crosses the universal measurement reference.

- Methane (CH_4)
- Water vapor (H_2O)
- Chlorofluorocarbons (CFCs)
- Carbon dioxide (CO_2)
- Nitrous oxide (N_2O)
- Others: _________

5. Main sources of GHG concentration (select two): Energy / Agriculture / Urban emissions / Transportation / Forest fires

6. Environmental sustainability pillars (select applicable): Economic development / Renewable energy / Social equity / Poverty reduction / Impact mitigation / Climate adaptation

7. Hazards resulting from global temperature increase (mark applicable): Sea level rise / Glacier reduction / Ocean warming / Hydrological cycle changes / Extreme weather events

8. Technological strategies to reduce GHG emissions (mark applicable): Energy efficiency / Renewables / Carbon capture / Digitalization / Sustainable transport / Industrial innovation / Offsetting / Education

9. Individual/family contributions to sustainability (mark applicable): Energy saving / Household efficiency / Water conservation / Renewables / Education / Local policies / Sustainable transport / Waste reduction

10. Consequences of climate change (mark applicable): Extreme weather / Biodiversity loss / Resource depletion / Socioeconomic effects / Public health impacts

11. Measures to control extreme climatic hazards (mark applicable): Cleaning rivers/canals / Tree pruning / Animal protection / Street cleaning / Housing in safe areas / Elevated housing / Protective barriers / Rainwater cisterns / Coastal vegetation / Wetland protection / Deep-rooted trees / Glass protection

12. Frequent actions to reduce environmental impact (mark applicable): Waste reduction / Waste separation / Walking to work / Reduce AC use / Turn off lights / Disconnect devices / Avoid standby mode / Energy-saving bulbs

13. Community priorities to mitigate CC (rank in order): Electricity price increase / Water price increase / Sustainable agriculture / Soil erosion reduction / Reforestation / Mangrove restoration / Reef restoration

14. Origin of climate change (scientific evidence): Human activity only / Human + natural variability / Natural variability only

15. Catastrophic potential of extreme events (mark one per case):

- Floods: Low / Medium / High
- Cyclones: Low / Medium / High
- Landslides: Low / Medium / High
- Heavy rainfall: Low / Medium / High

16. Immediacy of consequences (mark one per case):

- Floods: Slow / Medium / Rapid evolution
- Cyclones: Slow / Medium / Rapid evolution
- Landslides: Slow / Medium / Rapid evolution
- Heavy rainfall: Slow / Medium / Rapid evolution

17. Memories of destructive climatic phenomena: None / Clear / Vague

18. Psychological/emotional or physical impact of extreme events: Low / Medium / High

19. Recommended practices upon meteorological warnings (mark applicable): Canned food storage / Radio & flashlight / Roof & window repair / Potable water storage / Debris removal / Drain cleaning

20. Can consequences of climatic phenomena be reversed? No / Partially / Totally

21. Personal memories of victims of climatic phenomena: None / Some / Numerous

22. Governmental roles in reducing energy-related impacts (mark applicable): Fossil fuel replacement / Renewable energy use / Circular economy / Energy efficiency / Standards application / Forest management / Technology transfer / Efficient technologies / Transmission loss reduction / Climate finance

23. National actions to address climate crisis (mark applicable):

- **Energy:** Solar/wind use / Energy saving / Stop polluting fuels

- **Economy:** Green investment / Product transparency / Polluter pays
- **Transport:** Clean vehicles / Clean freight / Urban design
- **Food Security:** Climate-friendly agriculture / Food waste reduction / Plant-based diets
- **Protection of People:** Early warning systems / Civil defense / Affordable insurance / Infrastructure & nature conservation
- **Protection of Nature:** Forest conservation / Healthy oceans / Support local communities

24. Should affected communities receive material/financial support? No / According to impact / Beyond impact

25. Adequacy of beneficiaries' use of support: Inadequate / Partially adequate / Fully adequate / Not authorities' responsibility

26. Lifestyle changes for sustainability (mark one): Reduce impacts sacrificing comfort / Balance comfort with impact reduction / Preserve comfort without reducing impacts

27. Means of informing communities about CC initiatives (mark applicable): TV / Radio / Digital networks / Personal communication / Training / Family

**28. Additional topics of interest for risk perception or climate disaster management (please specify): ___________

Thank you very much for your participation.
